# Supplementary material for: Essential Amino Acid Starvation-Induced Oxidative Stress Causes DNA Damage and Apoptosis in Murine Osteoblast-like Cells
Source: Int J Mol Sci. 2023 Oct 18;24(20):15314. doi: 10.3390/ijms242015314 (PMC10607495; doi:10.3390/ijms242015314)

Figure S1 Original, unedited western blot image (p53 for Fig 3F, on the panel below)

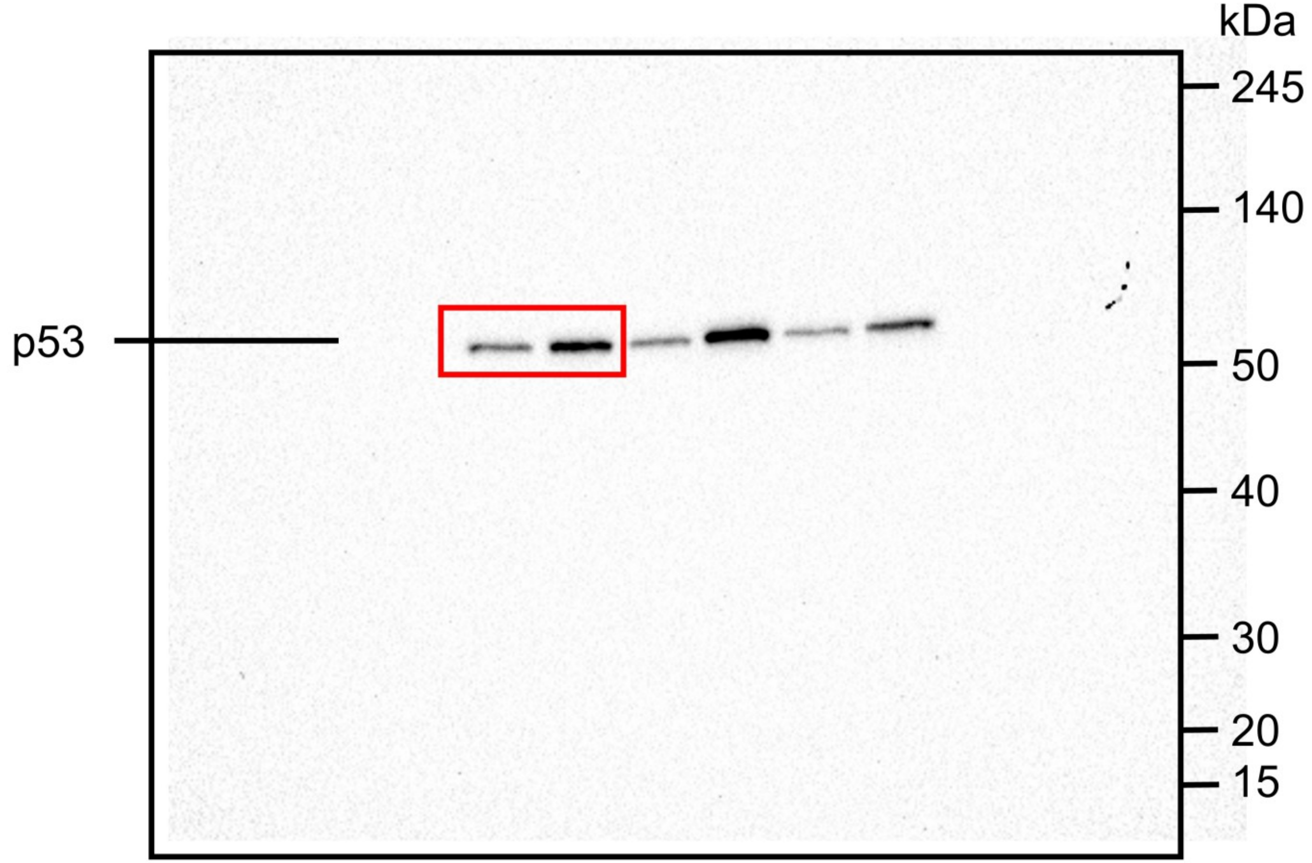

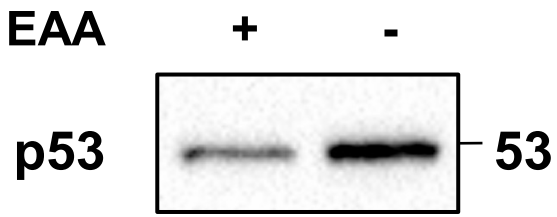

Figure S2 Original, unedited western blot image (Bax for Fig 3F, on the panel below)

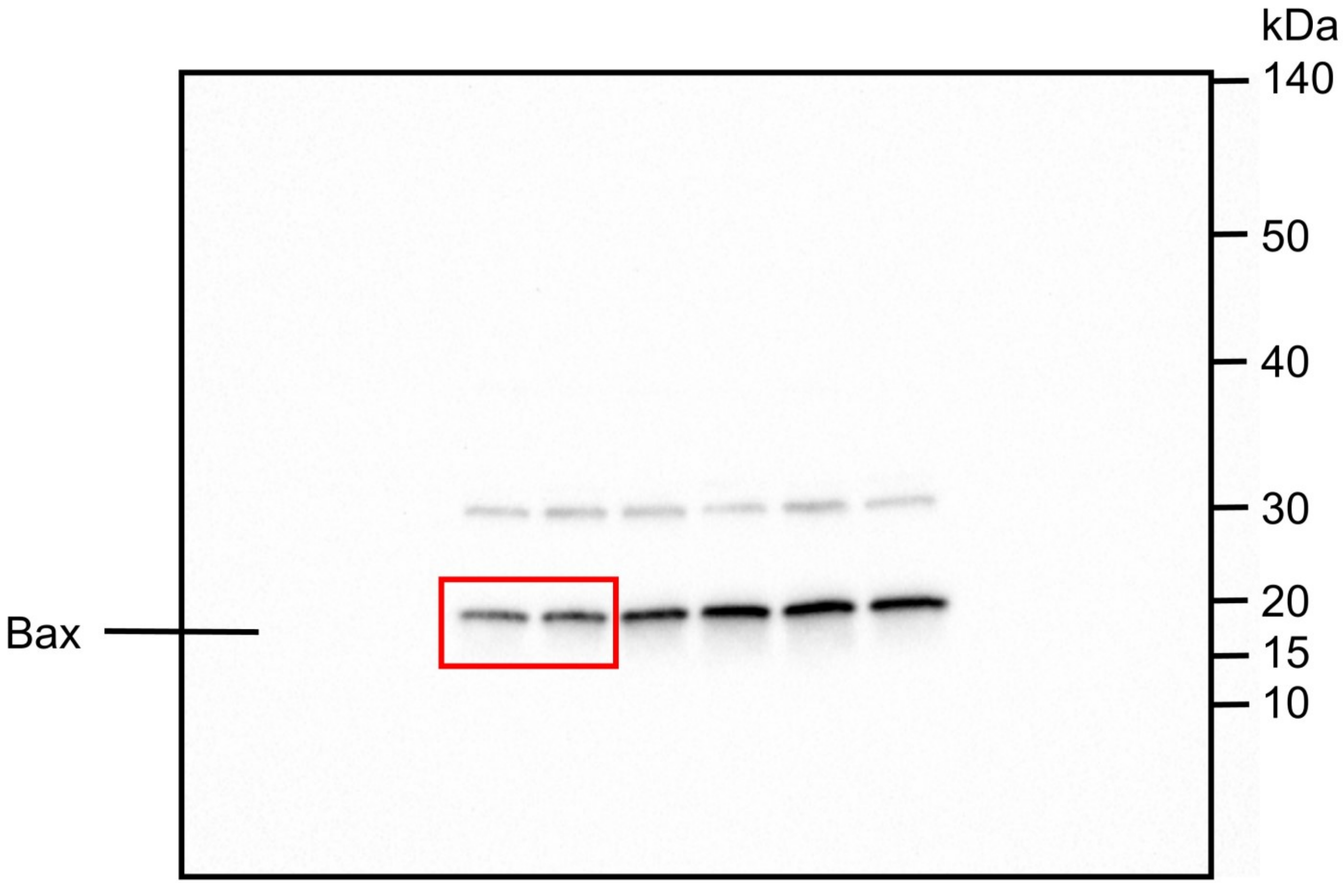

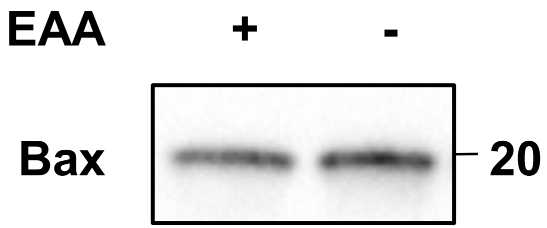

Figure S3 Original, unedited western blot image (Caspase-3 for Fig 3F, on the panel below)

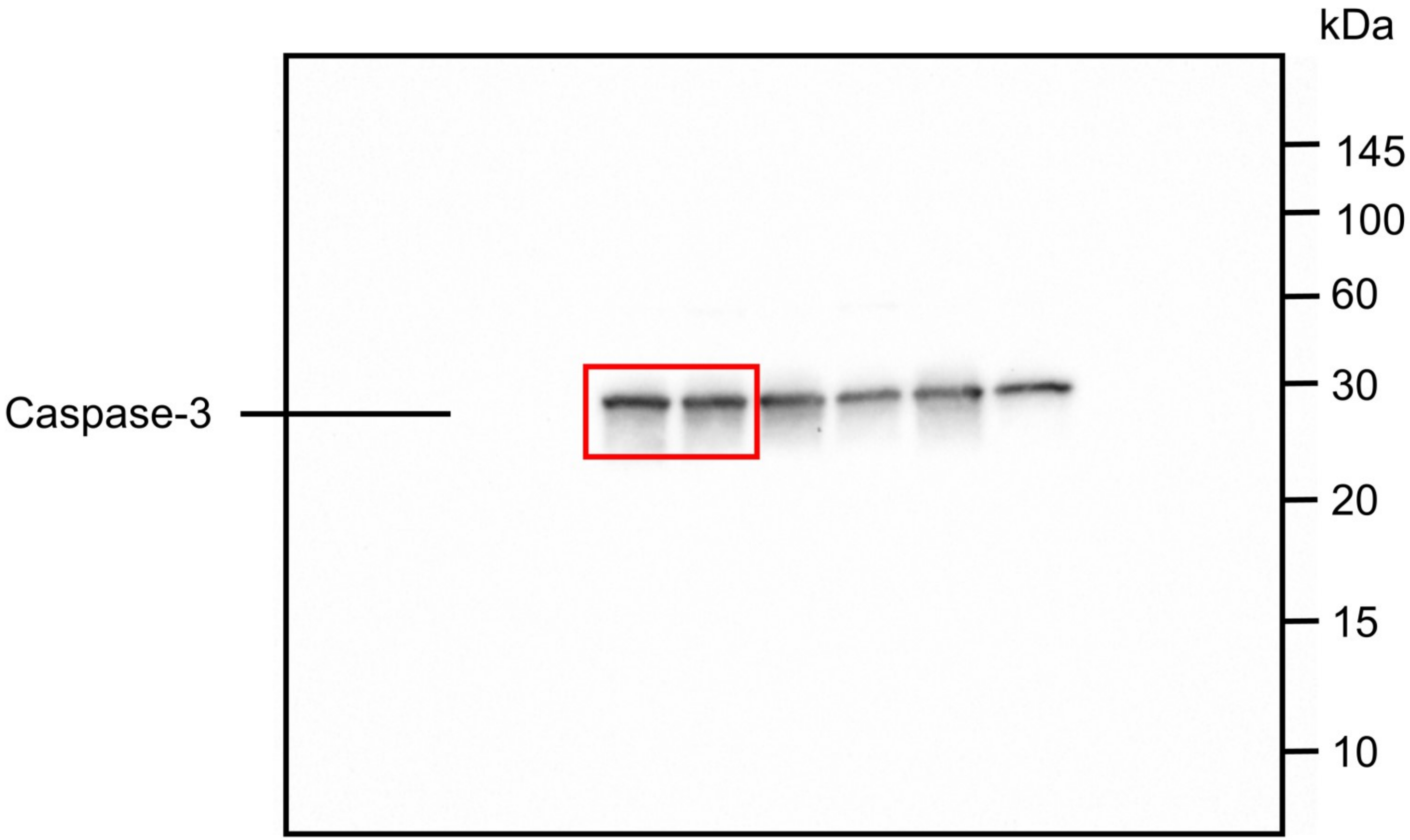

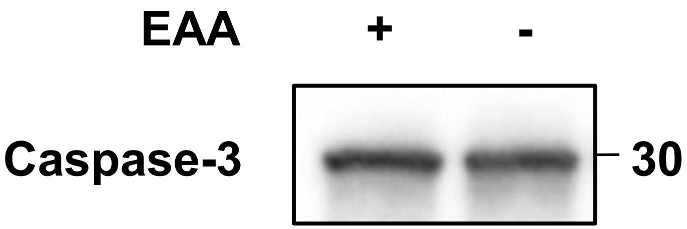

Figure S4 Original, unedited western blot image (Cleaved Caspase-3 for Fig 3F, on the panel below)

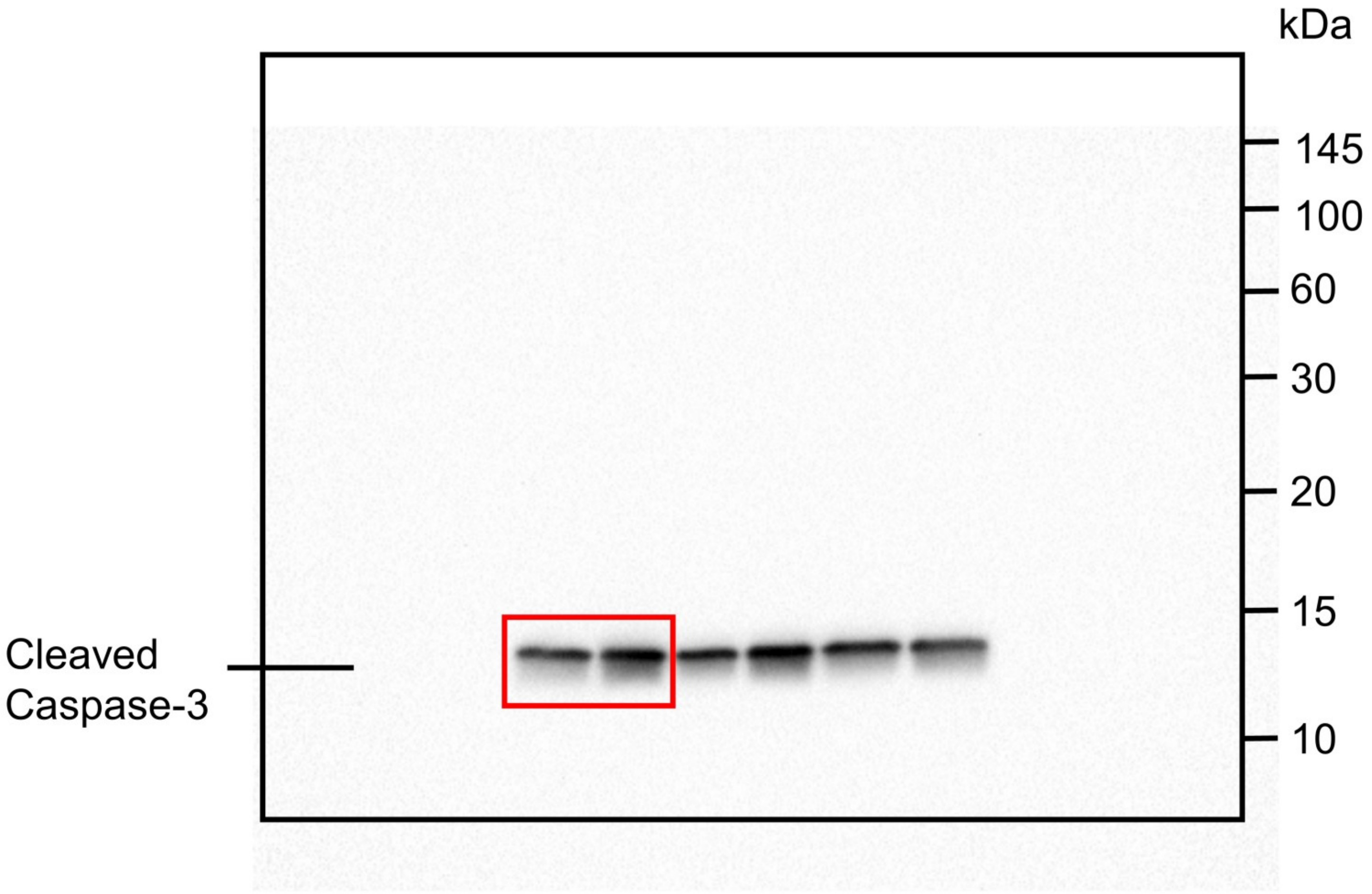

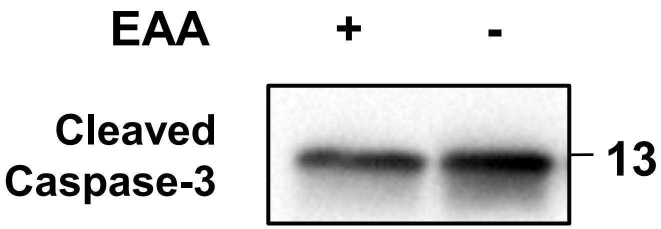

Figure S5 Original, unedited western blot image
(β-actin for Fig 3F, on the panel below, same menbrane as p53 and bax.)

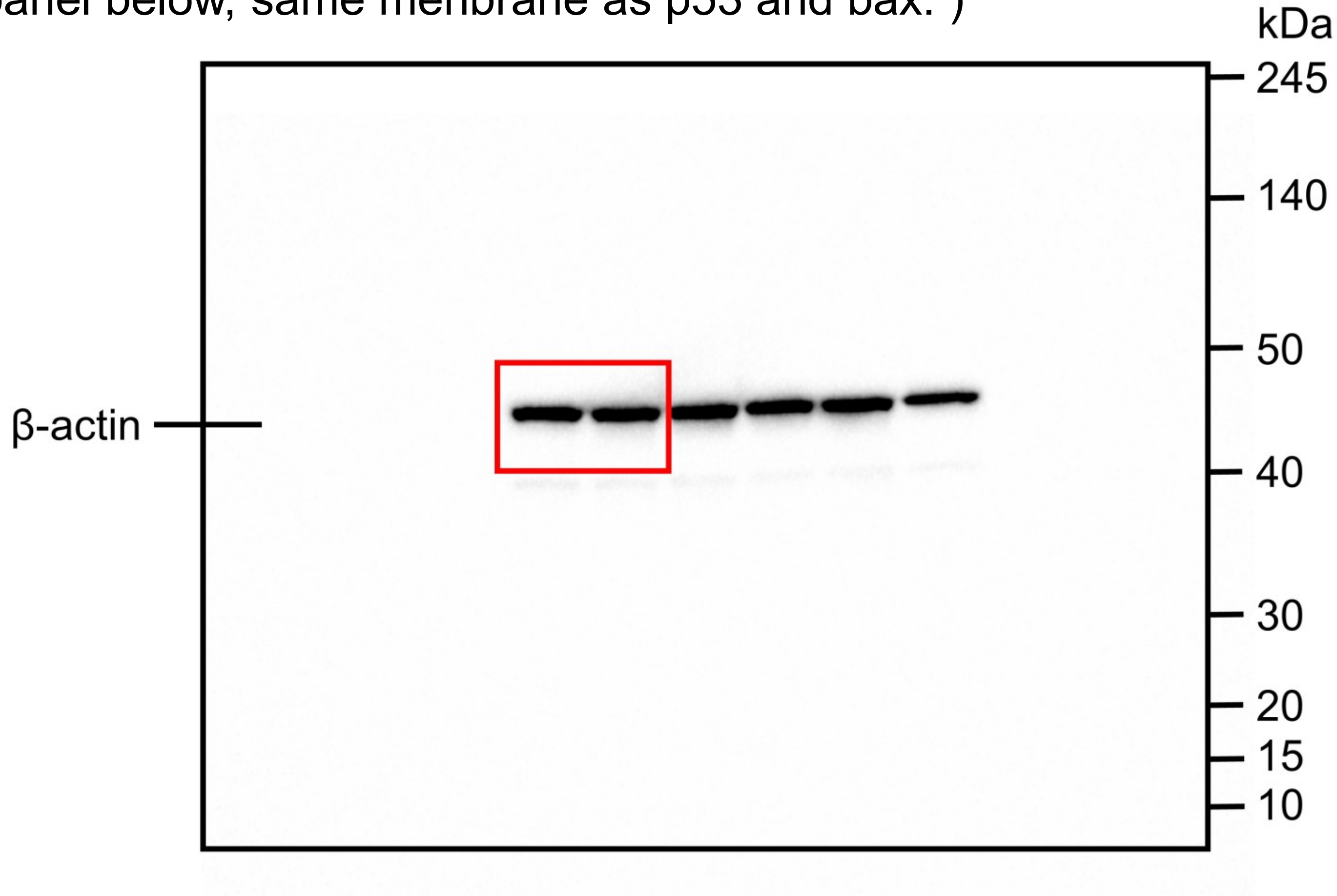

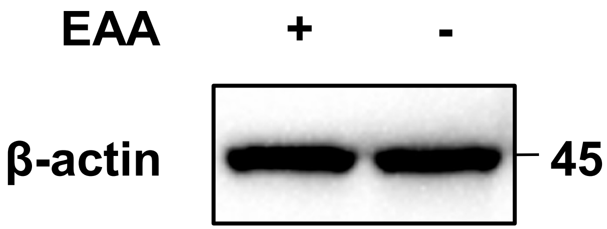

Figure S5.2 Original, unedited western blot image
(β-actin. Same menbrane as Caspase-3 and Cleaved Caspase-3.)

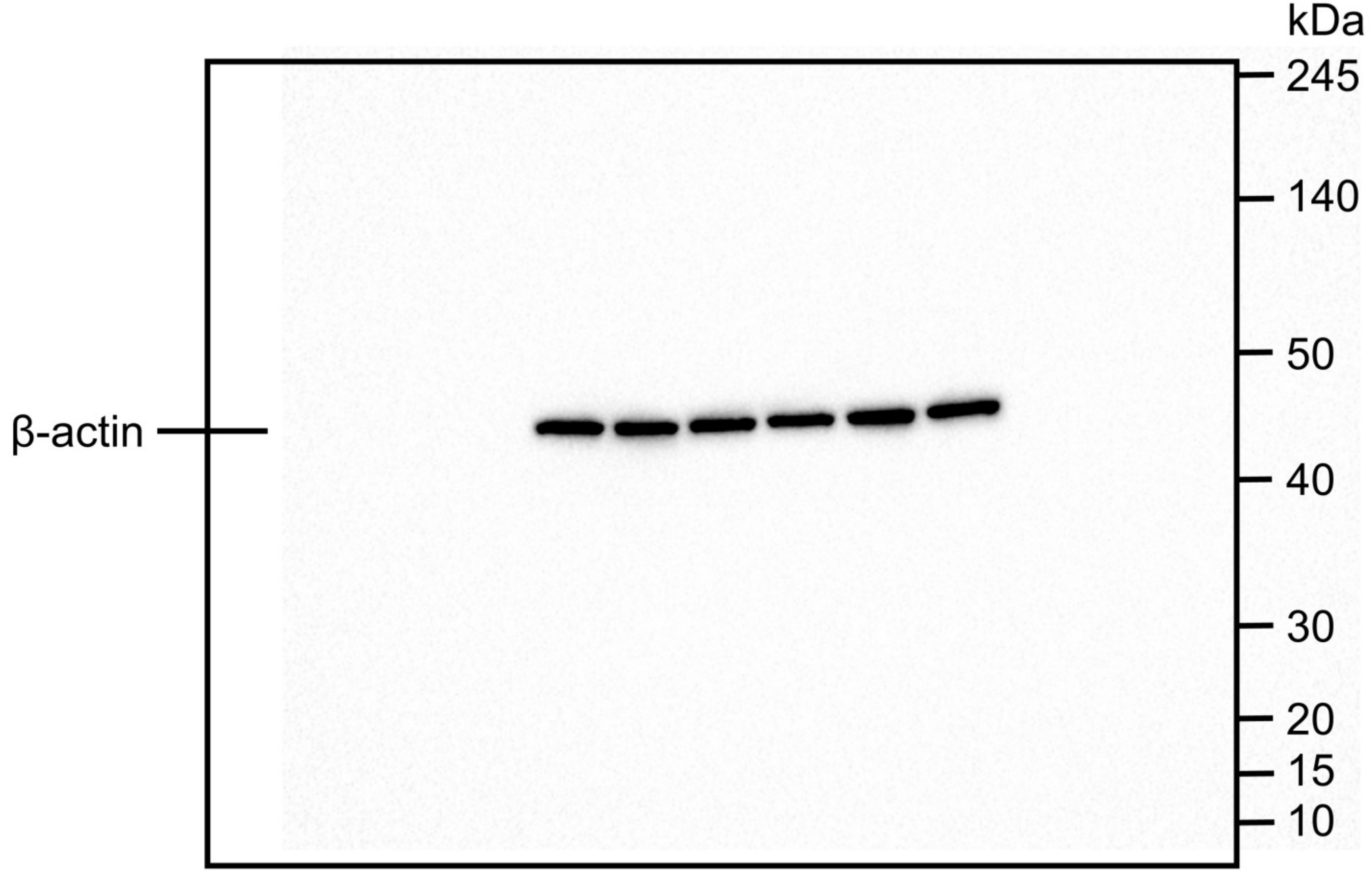

Figure S6 Original, unedited western blot image (p53 for Fig 6G, on the panel below)

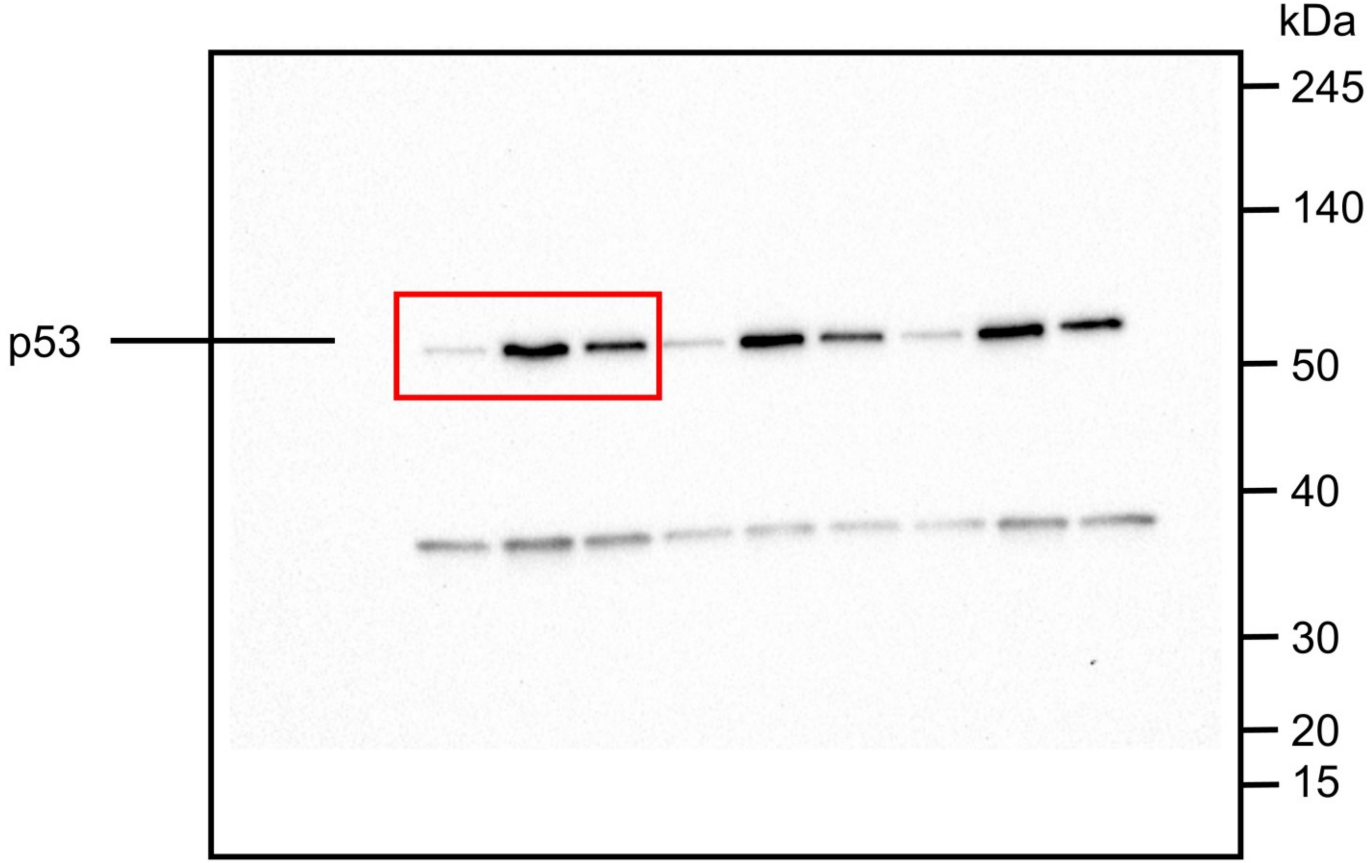

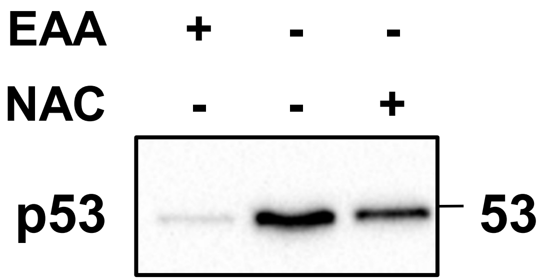

Figure S7 Original, unedited western blot image (Bax for Fig 6G, on the panel below)

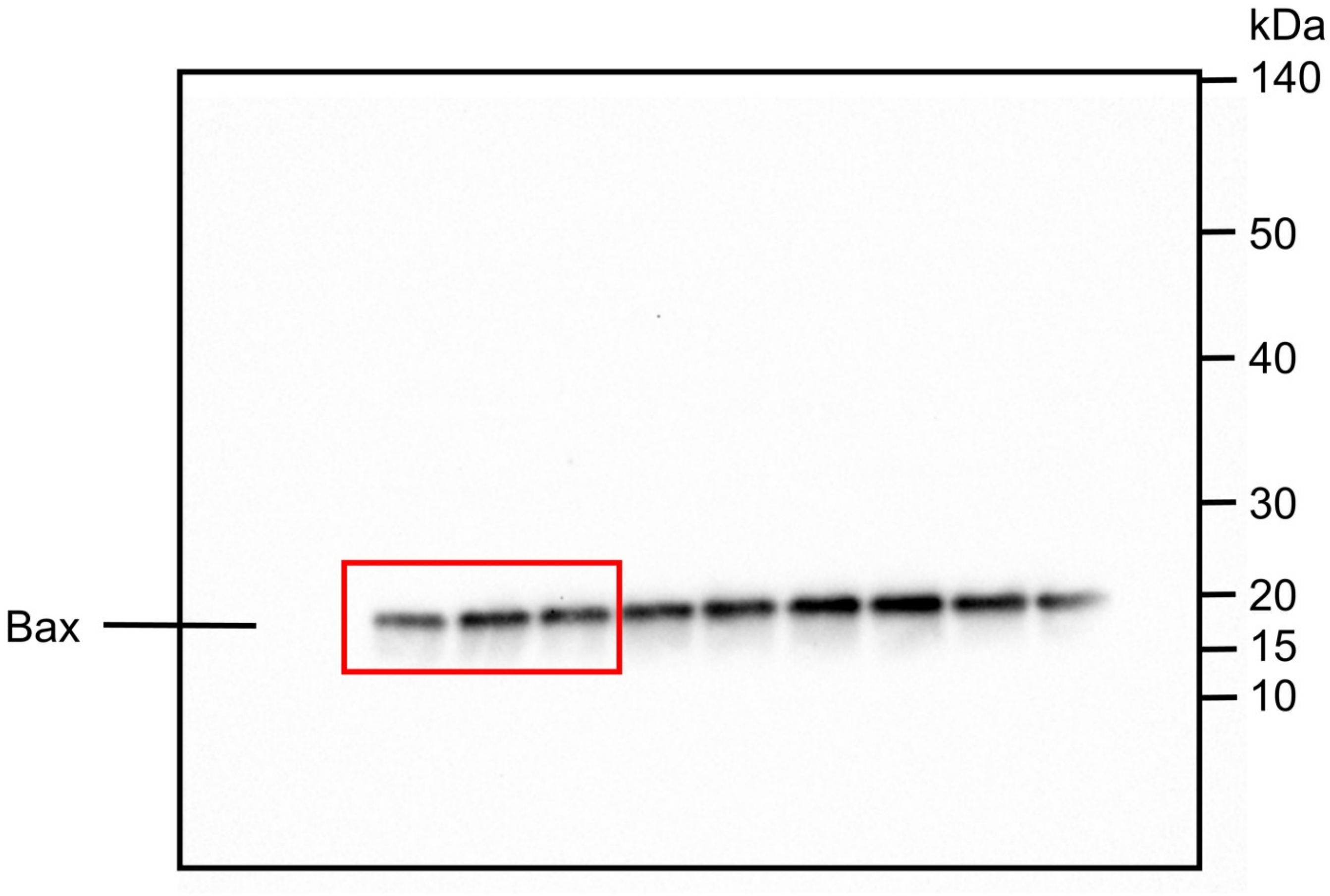

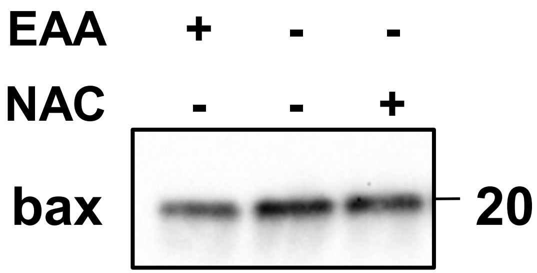

Figure S8 Original, unedited western blot image (Caspase-3 for Fig 6G, on the panel below)

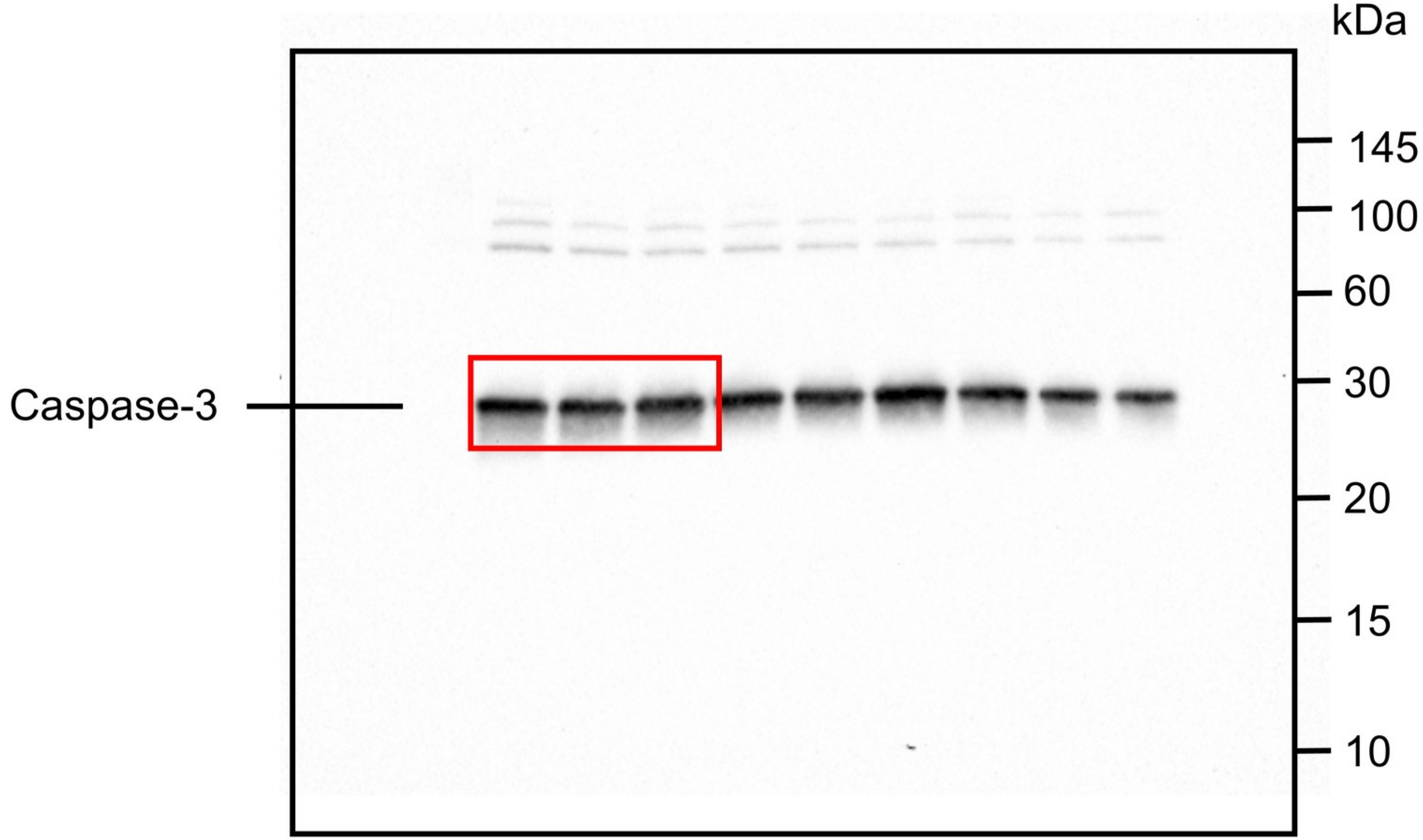

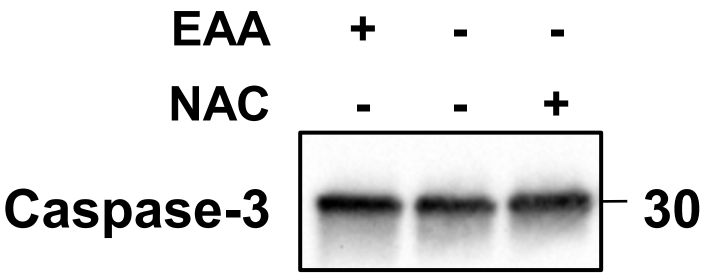

Figure S9 Original, unedited western blot image (Cleaved Caspase-3 for Fig 6G, on the panel below)

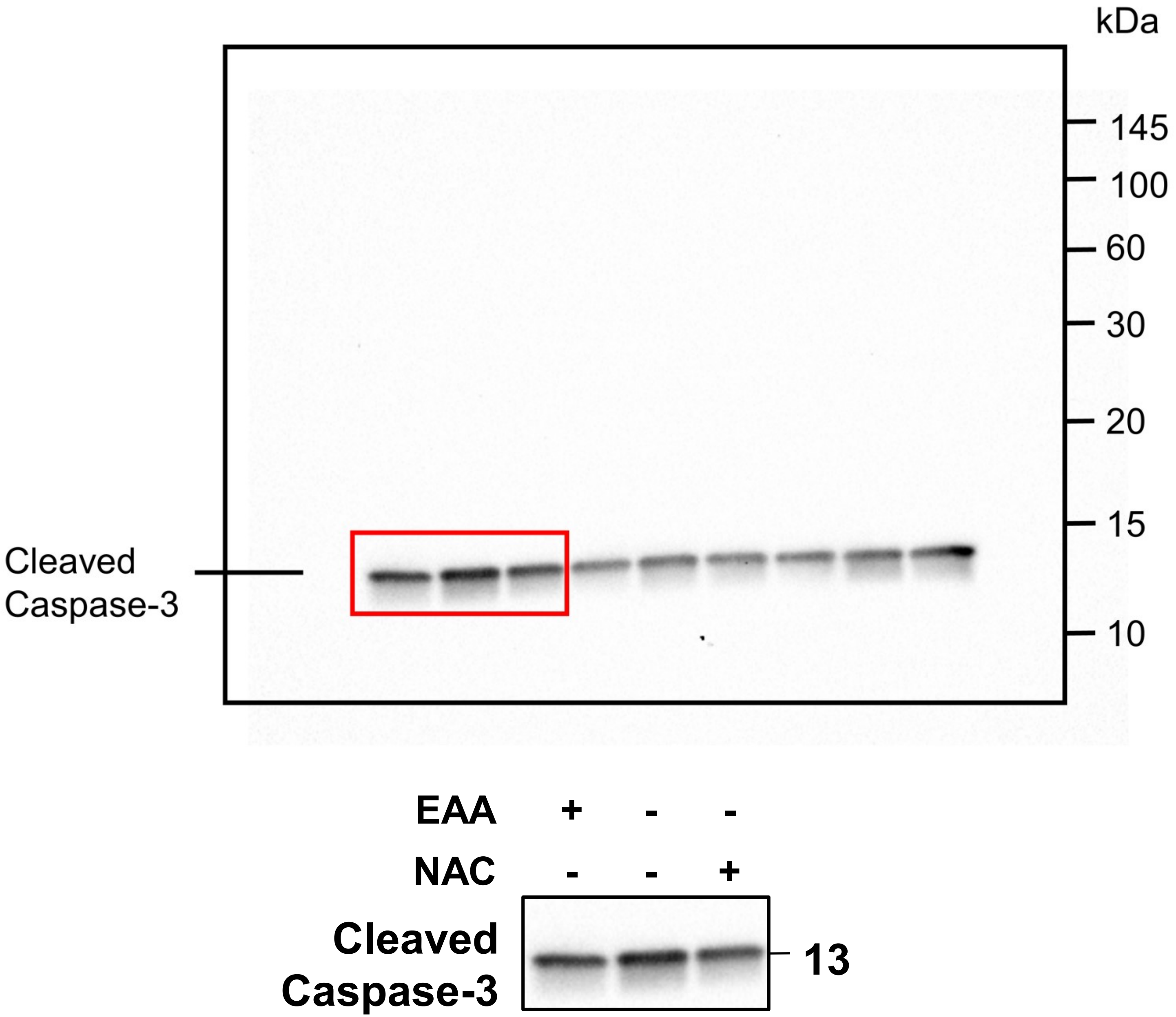

Figure S10 Original, unedited western blot image
(β-actin for Fig 6G, on the panel below, same menbrane as p53 and bax.)

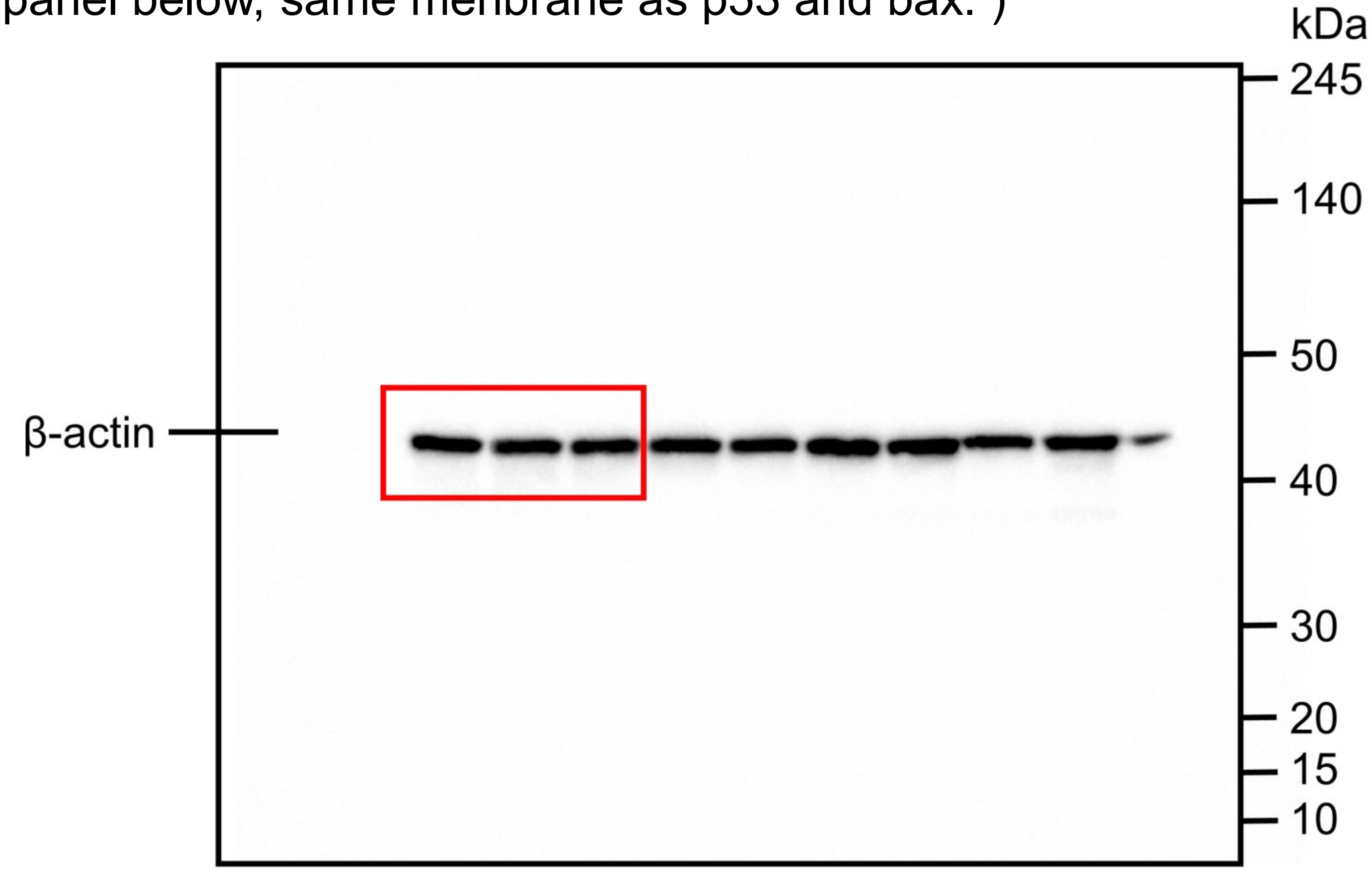

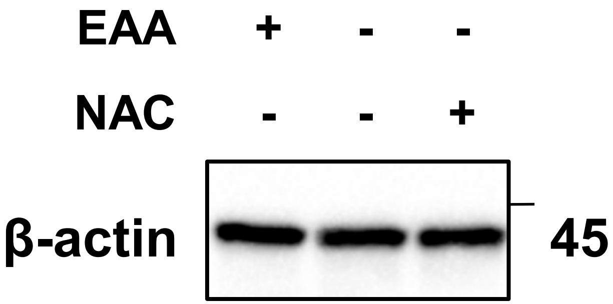

Figure S10.2 Original, unedited western blot image
(β-actin. Same menbrane as Caspase-3 and Cleaved Caspase-3.)

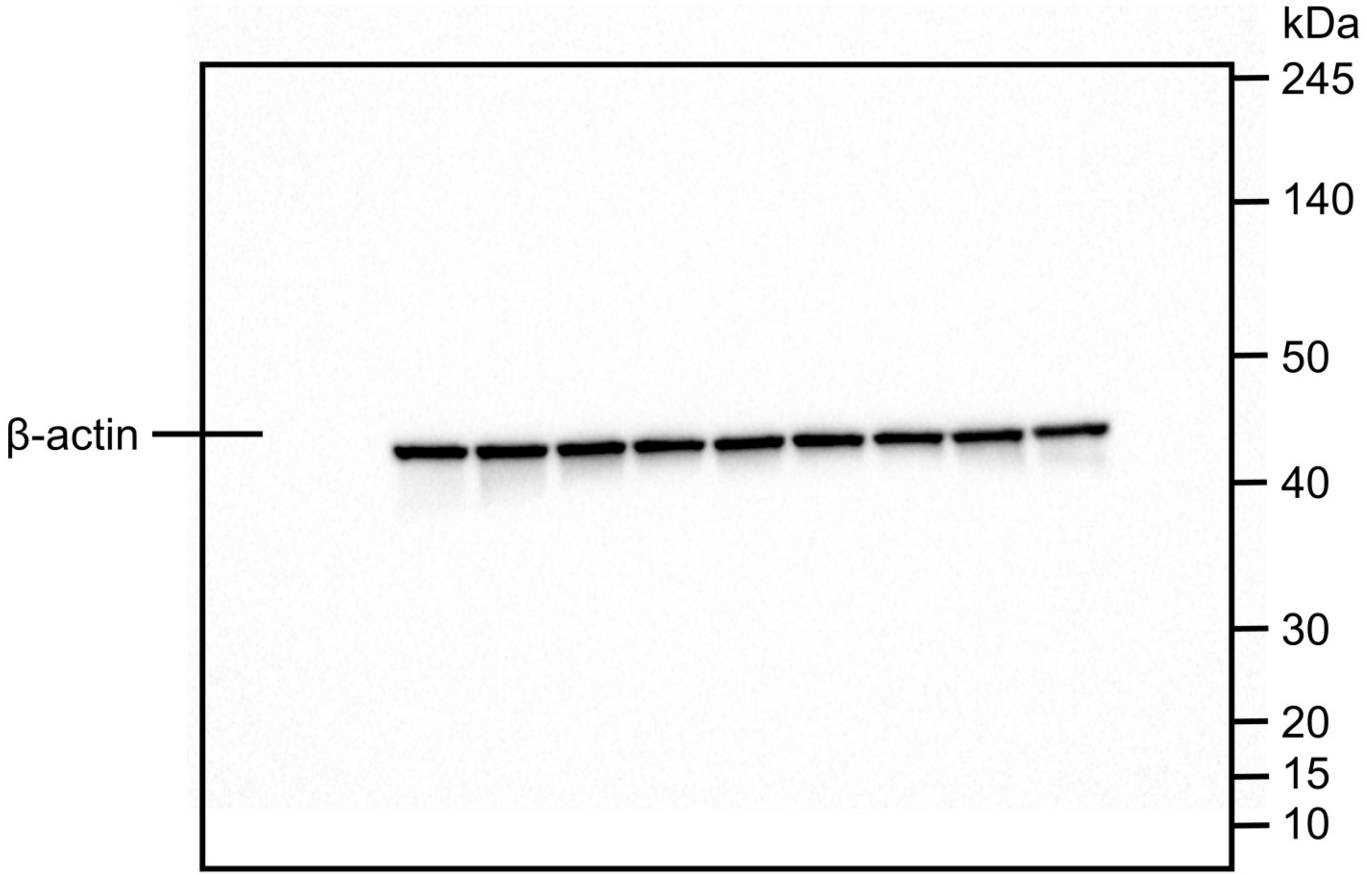

Supplement: Supplementary file 1 [file ijms-24-15314-s001.zip › ijms-2651008-supplementary.pdf]
